# Stimuli-Responsive Lyotropic Liquid Crystalline Nanosystems with Incorporated Poly(2-Dimethylamino Ethyl Methacrylate)-b-Poly(Lauryl Methacrylate) Amphiphilic Block Copolymer

**DOI:** 10.3390/polym11091400

**Published:** 2019-08-26

**Authors:** Maria Chountoulesi, Natassa Pippa, Varvara Chrysostomou, Stergios Pispas, Evangelia D. Chrysina, Aleksander Forys, Lukasz Otulakowski, Barbara Trzebicka, Costas Demetzos

**Affiliations:** 1 Section of Pharmaceutical Technology, Department of Pharmacy, School of Health Sciences, National and Kapodistrian University of Athens, Panepistimioupolis Zografou, 15771 Athens, Greece; 2 Theoretical and Physical Chemistry Institute, National Hellenic Research Foundation, 48 Vassileos Constantinou Avenue, 11635 Athens, Greece; 3 Institute of Chemical Biology, National Hellenic Research Foundation, 48 Vassileos Constantinou Avenue, 11635 Athens, Greece; 4 Centre of Polymer and Carbon Materials, Polish Academy of Sciences, 34 ul. M. Curie-Skłodowskiej, 34, 41-819 Zabrze, Poland

**Keywords:** lyotropic liquid crystals, poly(2-dimethylaminoethyl methacrylate), stimuli-responsiveness, block copolymer, drug delivery nanosystems, cryo-TEM

## Abstract

There is an emerging need to evolve the conventional lyotropic liquid crystalline nanoparticles to advanced stimuli-responsive, therapeutic nanosystems with upgraded functionality. Towards this effort, typically used stabilizers, such as Pluronics^®^, can be combined or replaced by smart, stimuli-responsive block copolymers. The aim of this study is to incorporate the stimuli-responsive amphiphilic block copolymer poly(2-(dimethylamino)ethyl methacrylate)-b-poly(lauryl methacrylate) (PDMAEMA-b-PLMA) as a stabilizer in lipidic liquid crystalline nanoparticles, in order to provide steric stabilization and simultaneous stimuli-responsiveness. The physicochemical and morphological characteristics of the prepared nanosystems were investigated by light scattering techniques, cryogenic-transmission electron microscopy (cryo-TEM), X-ray diffraction (XRD) and fluorescence spectroscopy. The PDMAEMA-b-PLMA, either individually or combined with Poloxamer 407, exhibited different modes of stabilization depending on the lipid used. Due to the protonation ability of PDMAEMA blocks in acidic pH, the nanoparticles exhibited high positive charge, as well as pH-responsive charge conversion, which can be exploited towards pharmaceutical applications. The ionic strength, temperature and serum proteins influenced the physicochemical behavior of the nanoparticles, while the polymer concentration differentiated their morphology; their micropolarity and microfluidity were also evaluated. The proposed liquid crystalline nanosystems can be considered as novel and attractive pH-responsive drug and gene delivery nanocarriers due to their polycationic content.

## 1. Introduction

Non-lamellar, lyotropic liquid crystalline nanoparticles, such as cubosomes and hexosomes, are proved to be ideal therapeutic nanosystems, allowing sustained content release and bearing tunable structural characteristics [1,2,3]. They are usually prepared from the lipids glyceryl monooleate (GMO) or phytantriol (PHYT) and require the presence of amphiphilic block copolymers, known as stabilizers, in order for stable colloidal dispersions of nanoparticles to be achieved. Stabilization by the Pluronics^®^ triblock copolymers, usually by F-127 (Poloxamer P407), is the most well investigated [2,3,4,5]. 

Although P407 is commercially available and provides efficient steric, long term stabilization, as well as biocompatibility, it lacks functional targeting groups [6]. Thus, P407 liquid crystalline formulation’s lack stimuli-responsive mechanisms that would enhance their advantages. More particularly, the stimuli-responsive nanosystems, employing a great variety of smart stimuli-responsive polymers, are able to reach specific intracellular locations, overcome the intermediate barriers, retain prolonged circulation time and promote targeted, controlled drug/gene release. The smartness of the stimuli-responsive polymers and their resultant nanosystems is based on their response to environmental stimuli, such as pH, temperature, ionic strength, etc., by changing their physicochemical and morphological characteristics [7,8,9,10]. For example, the stimuli-responsive nanosystems take advantage of the altered pH/temperature values exhibited at the pathological tissues. Moreover, the pH gradients of the intracellular level can be exploited upon endosomal and lysosomal escape. The ability of endosomal and lysosomal escape is considered to be a crucial advantage for nanosystems towards the achievement of more effective drug and gene delivery exactly to the targeted site into the cell and so higher bioavailability [7,8,9,10,11,12,13]. 

Although there are several literature examples about stimuli-responsive liquid crystalline nanosystems [14,15,16,17], there are not many proposed polymeric stimuli-responsive stabilizers [18]. The utilization of alternative block copolymers as stabilizers for the liquid crystalline nanoparticles is a new field that has recently been gaining momentum. The basic scope of this approach is the generation of multifunctional, active targeting liquid crystalline carriers, such as the stimuli-responsive ones. However, the choice of a suitable stabilizer at the right concentration is a difficult process that depends on the chemical structure, molecular weight and hydrophobic–hydrophilic ratio of the chosen polymer [6,18,19]. 

Poly(2-(N,N-dimethylamino) ethyl methacrylate) (PDMAEMA) is a weak cationic polyelectrolyte, frequently used as a biomaterial, which exhibits both pH- and temperature-responsive behavior. It contains an ionizable tertiary amino group, which is protonated under acidic environmental conditions. The polymer exhibits a conversion from hydrophilic to hydrophobic character under pH and temperature increase, because of deprotonation and polymer–polymer interactions [9,11,12,20,21,22]. Furthermore, due to its intrinsic cationic charge, PDMAEMA is considered to be suitable for gene delivery applications, through electrostatic complexation with the negatively charged nucleic acids [23,24,25]. Regarding its temperature response, PDMAEMA presents a lower critical solution temperature (LCST) in water in the range 40–50 °C at pH ca. 7 [21,26,27].

The goal of the present study is the development of advanced stimuli-responsive liquid crystalline nanoparticles. Moreover, the utilization of a block copolymer that presents a dual role, acting as a stabilizer and at the same time providing the desired stimuli-responsiveness, is proposed. In this context, we prepared liquid crystalline nanoparticles, incorporating an amphiphilic block copolymer, consisting of the hydrophilic stimuli-responsive PDMAEMA and the hydrophobic poly(lauryl methacrylate) (PLMA), being suitable for novel drug and gene delivery nanosystems. To the best of the authors’ knowledge, this is the first report on liquid crystalline nanoparticles employing polycations of PDMAEMA. This approach opens new paths towards drug and gene delivery applications.

In particular, a series of systems containing GMO or PHYT lipid and two different amounts of PDMAEMA-b-PLMA were prepared. Another, more complex formulation was also prepared, incorporating equal amounts of both PDMAEMA-b-PLMA and P407. A gamut of light scattering techniques, including static, dynamic and electrophoretic light scattering, were applied for the morphological and physicochemical characterization of the nanoparticles. The prepared systems were also studied by X-ray diffraction (XRD) and their morphology was verified by cryogenic transmission electron microscopy (cryo-TEM). Since PDMAEMA possesses a dual-stimuli responsive character, we monitored the response of the nanosystems to both pH and temperature alterations. Additionally, we studied the effect of the serum proteins and the ionic strength on their physicochemical behaviour. Lastly, two important microenvironmental parameters of the nanoparticles, namely micropolarity and microfluidity, were investigated by fluorescence spectroscopy.

We should mention that the present study is a continuation of our recently published work about liquid crystalline nanosystems [28]. Our further goal is the upgrade of the already existing liquid crystalline nanosystems to advanced therapeutic nanosystems, by exploiting smart biomaterials. Thus, the innovation of the present study, as well as the progress in comparison to our previous work, is the incorporation of the aforementioned stimuli-responsive amphiphilic polycationic copolymer within the liquid crystalline nanosystems.

## 2. Materials and Methods

### 2.1. Materials

The lipids that were employed to prepare liquid crystalline formulations were phytantriol (PHYT, 3,7,11,15-tetramethyl-1,2,3-hexadecanetriol), which was purchased from DSM Nutritional Products Ltd., and glyceryl monooleate (Monomuls^®^ 90-O18) [GMO, 1-(cis-9-octadecenoyl)-rac-glycerol], which was purchased from BASF (Düsseldorf, Germany); both were used without further purification. According to the literature, where the Monomuls^®^ 90-O18 is used, it presents a monoglyceride content of 95.7%(w/w) and oleic acid content of 90%(w/w), being suitable for the preparation of cubic or hexagonal liquid crystalline phases [29,30,31,32] and behaving like that of pure GMO component [33]. According to the certificate analysis of BASF, the acid value of Monomuls^®^ 90-O18 is 1.9 mg KOH/g. The poly(2-(dimethylamino)ethyl methacrylate)-b-poly(lauryl methacrylate) (PDMAEMA-b-PLMA) amphiphilic diblock copolymer was synthesized by RAFT polymerization methodology, in PDMAEMA:PLMA 69.7:30.3 weight composition [21]. The molar mass (*M_w_*) of the diblock copolymer, determined by size exclusion chromatography (SEC), equates to 9600 g/mol and its polydispersity (PDI = M_w_/M_n_), as determined by SEC, is 1.17. Pluronic^®^ F-127 (Poloxamer P407) (PEO_98_-PPO_67_-PEO_98_), with an average molar weight of 12,600 g/mol, was acquired from Sigma-Aldrich Chemical Co. (Merck, NJ, USA). All formulations were prepared in HPLC-grade water. Chloroform, methanol and acetone were of analytical grade and purchased from Sigma-Aldrich Chemical Co. Fetal bovine serum (FBS) was purchased from Gibco^®^. Pyrene was purchased from Sigma-Aldrich Chemical Co. and dissolved in appropriate concentration (1 mM) in acetone. Sodium chloride (NaCl) salt, citric acid, citrate sodium and Phosphate Buffered Saline (PBS) tablets were purchased from Sigma-Aldrich Chemical Co. and dissolved in appropriate concentrations in HPLC-grade water.

### 2.2. Methods

#### 2.2.1. Preparation of Liquid Crystalline Nanoparticles Dispersions

Block copolymer PDMAEMA-b-PLMA was used as the stabilizer either individually or in combination with P407. Three different weight ratios were prepared, namely, Lipid:PDMAEMA-b-PLMA 9:1, Lipid:PDMAEMA-b-PLMA 9:3 and Lipid:PDMAEMA-b-PLMA:P407 8:1:1 that correspond to three different concentrations of total stabilizer, namely 10%, 25% and 20% w/w. The lipid concentration of all the prepared systems was 20 mg/mL. The temperature used, during the preparation process of all the systems, was 45 °C and was chosen according to the temperature-concentration phase diagrams of each lipid in water, so cubosomal dispersions could be achieved. A temperature of 45 °C was the minimum that could be used without overtaking the LCST of the block copolymer.

All systems were prepared by top down method (TD), modified with an intermediate step of thin lipidic film preparation. More analytically, 200 mg of each lipid was fully dissolved in chloroform. PDMAEMA-b-PLMA block copolymer was fully dissolved in chloroform:methanol 9:1 (5 mg/mL w/v). Dissolved lipid and block copolymer were mixed in appropriate amounts and then transferred into a round flask connected to a rotary evaporator (Rotavapor R-114, Buchi, Switzerland). A vacuum was applied and the mixed lipid/block copolymer thin film was formed by slow removal of the solvent at 45 °C. Then, 10 mL of HPLC-grade water (pH = 6.0) was added to the flasks containing the lipid-block copolymer film. In the case of the 8:1:1 systems, the P407 was dissolved in the HPLC-grade water that was subsequently added to the film. The mixtures were sonicated in a bath sonicator for 30 min at 45 °C until a milky dispersion was formed, followed by three 5 min sonication cycles (amplitude 70, cycle 0,7), using an ultrasonicator (UP 200 S, DrHielsher GmbH, Berlin, Germany) and interrupted by two 5 min resting periods in order to prevent overheating of the samples. In some systems, acidification was required; this was conducted by adding drop-wise hydrochloric acid 1N until homogenous dispersion was achieved. The resultant dispersions were allowed to anneal for 30 min and then were transferred into vials and stored at room temperature.

#### 2.2.2. Physicochemical and Morphological Characterization of the Prepared Liquid Crystalline Dispersions

##### Dynamic, Static and Electrophoretic Light Scattering Techniques

The physicochemical behavior of the prepared nanosystems was evaluated by measuring the size (hydrodynamic radius *R_h_*, nm) and size distribution (polydispersity index, PDI) by utilizing dynamic light scattering (DLS). The ζ-potential (ζ-pot, mV) of the nanoparticles was measured by electrophoretic light scattering (ELS). The radius of gyration (*R_g_*, nm) was measured by static light scattering (SLS), in order for the *R_g_/R_h_* ratio to be estimated, which is indicative of the shape of the nanoassemblies. A total of 100 μL of aliquots were diluted 30-fold in HPLC grade water. The protocols used for DLS, ELS and SLS techniques are described in detail in our previous works [28,34,35] (See also SI). The *R_h_*, PDI and ζ-pot values of the nanoparticles were averaged from triplicate measurements and the results were reported as a mean ± standard deviation.

The effect of the pH of the medium was investigated by diluting 100 μL of aliquots 30-fold in three different pH media, namely, HPLC-grade water with pH = 6.0, phosphate buffered saline (PBS) with pH = 7.4, and citrate buffer with pH = 4.2. The samples were incubated at room temperature for 20 min and the DLS and ELS measurements were repeated as described. SLS measurement was repeated in citrate buffer too. The protocol of dissolution and incubation of the nanoparticle dispersion in different buffers exhibiting different pH values can easily display the potential pH-responsiveness of the systems [36,37,38].

The effect of serum proteins (fetal bovine serum, FBS), ionic strength (NaCl 0.10, 0.34, 0.51 N and phosphate buffered saline, PBS, 0.154 N) and temperature in the physicochemical behavior of the nanosystems was monitored by using DLS, SLS and ELS measurements, as described in our previous study [28] and also at SI.

##### Cryogenic Transmission Electron Microscopy (Cryo-TEM)

Cryogenic transmission electron microscopy (Cryo-TEM) micrographs were obtained using a Tecnai F20 TWIN microscope (FEI Company, Hillsboro, Oregon, USA) equipped with field emission gun, operating at an accelerating voltage of 200 kV. Images were recorded on the Eagle 4 k HS camera (FEI Company, USA) and processed with TIA software (FEI Company, Hillsboro, Oregon, USA). Specimen preparation was done by vitrification of the aqueous (HPLC-grade water) solutions on grids with holey carbon film (Quantifoil R 2/2; Quantifoil Micro Tools GmbH, Germany). Prior to use, the grids were activated for 15 s in oxygen plasma using a Femto plasma cleaner (Diener Electronic, Germany). Cryo-samples were prepared by applying a droplet (3 μL) of the solution to the grid, blotting with filter paper and immediately freezing it in liquid ethane using a fully automated blotting device, Vitrobot Mark IV (FEI Company, Hillsboro, Oregon, USA). After preparation, the vitrified specimens were kept in liquid nitrogen until they were inserted into a cryo-TEM-holder Gatan 626 (Gatan Inc., Pleasanton, California, USA) and analyzed in the TEM at −178 °C. Pictures were processed using ImageJ software.

##### X-ray Diffraction (XRD)

X-ray diffraction data collection and analysis was carried out on the six nanodispersions prepared, as also described in our recent study [28]. The in-house X-ray generator (manufactured in Poland by Agilent Technologies (Oxford diffraction) with microfocus sealed tube, CuKα radiation, λ = 1.54 Å, equipped with a kappa goniometer and a charged coupled detector, diameter 135 mm) installed at the National Hellenic Research Foundation (NHRF) was used. The X-rays were generated at power settings of 50 kV and 0.8 mA. The samples were mounted on capillaries and exposed to X-rays for 300 sec at a specimen-to-detector distance of 54.8 cm. The 2D diffraction plots for relative intensity vs. 2θ were calculated and peak assignment was performed using CrysAlisPro software. Further analysis for depicting the collected data in graphical schemes was carried out using Microsoft Excel software.

##### Fluorescence Spectroscopy

Fluorescence spectroscopy was applied in order to extract some qualitative information about the internal microenvironment of the prepared nanoparticles (micropolarity and microfluidity), by using pyrene as hydrophobic probe. The measurements were carried out at two different temperatures 25 °C and 45 °C, in two different pH media (HPLC-grade water with pH = 6.0 and citrate buffer with pH = 4.2). The protocol is described in detail by Chountoulesi et al. [28] and Pippa et al. [34,35] (See also SI).

## 3. Results and Discussion

### 3.1. Results from Light Scattering Techniques

#### 3.1.1. PDMAEMA-b-PLMA Block Copolymer Performing as Stabilizer

**Figure 1:**
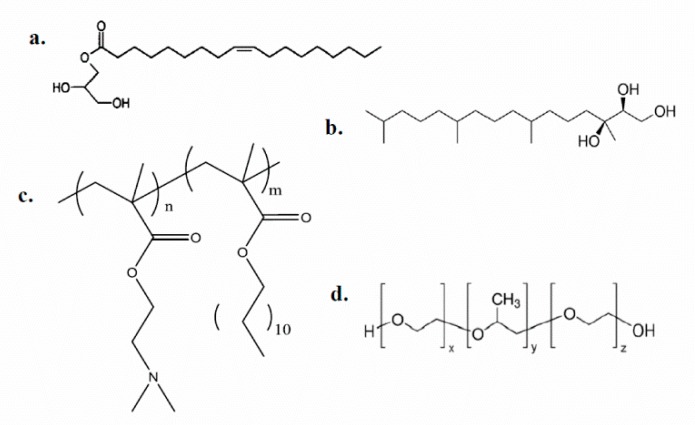
Chemical structures of **a.** glyceryl monooleate (GMO), **b.** phytantriol (PHYT), **c.** poly(2-(dimethylamino)ethyl methacrylate)-b-poly(lauryl methacrylate) (PDMAEMA-b-PLMA), **d.** Poloxamer P407.

The chemical structures of all the materials utilized are illustrated in Figure 1. The prepared systems presented many differences in the physicochemical behavior, being obvious from the first stages of the preparation process till their afterward lifecycle. A minimum P407 concentration of 10% w/w vs. lipid (i.e., 9:1 lipid-to-P407) is required to produce aggregate-free cubosomes [39]. Thus, we tried the lowest required stabilizer ratio, replacing the P407 by PDMAEMA-b-PLMA, in order to investigate the ability of the PDMAEMA-b-PLMA copolymer to act as stabilizing agent. To the best of our knowledge, as it was the first time that a copolymer of PDMAEMA and PLMA is employed as a stabilizer in liquid crystalline nanosystems and its behaviour was quite unpredictable, we chose a PDMAEMA-b-PLMA block copolymer that possesses similar hydrophilic-to-hydrophobic ratio (69.7:30.3) with P407 (70% PEO as reported by Chong et al. [40]) as a starting common point. After all, our intention was to use a block copolymer that, apart from its pH-responsiveness, is also able to co-assemble with the lipids and stabilize them towards the formation of liquid crystalline nanoparticles. While we have already studied in detail the lipid:P407 systems in our previous investigation [28], we used these results as controls for comparison reasons and we did not repeat the preparation of those systems in the present work.

PDMAEMA-b-PLMA had difficulties in stabilizing the GMO:PDMAEMA-b-PLMA 9:1 and 9:3 systems, while a step-wise acidification was required, in order to achieve homogenous dispersions, with final pH values as presented in Table 1. The more acidic medium caused higher protonation degree of the PDMAEMA block and increased its hydrophilicity, so it eventually made the initial lipid-polymer aggregates to be homogenously dispersed. After all, a higher hydrophilic-to-hydrophobic ratio of the polymer promotes a more efficient stabilization [40]. As Chong et al. [40,41] describe, the longer hydrophilic polymeric block yields greater entropic effect and a more sufficient stabilizing effect. Thus, in the case of the tricomponent GMO:PDMAEMA-b-PLMA:P407 system, the longer length of the PEO block of P407 compared to the PDMAEMA block, may have contributed to its stabilization without further acidification. Contrariwise, all PHYT systems were more easily prepared without any acidification (Table 1). As Zhai et al. [19] and Chong et al. [41] have described, the “kink” being presented in the unsaturated oleyl chain of monoolein due to its double bond, affects the affinity of binding with the hydrophobic part of the novel stabilizers. Furthermore, in our previous investigation [28], we used exactly the same materials sources of both GMO and PHYT lipids. Neither GMO:P407 nor PHYT:P407 systems required acidification in order to be successfully dispersed. Taking into account the above fact, we assume that the necessity of acidification is attributed to the presence of PDMAEMA-b-PLMA and not to the rest of the materials. Regarding the macroscopic examination (Table 1), the milky opaque appearance of the dispersions, except from GMO: PDMAEMA-b-PLMA 9:3, is an indication of a well-defined liquid crystalline structure. Analytically, the well-defined geometric segregation of the lipid and water domains of the cubic and hexagonal phases causes a strong Rayleigh scattering to visible light, yielding an opaque external appearance [39,42].

The physicochemical characteristics of the prepared nanosystems at the day of preparation (t = 0 days) are summarized in Table 2. The size difference between PHYT and GMO systems may reflect a different way of interaction of PDMAEMA-b-PLMA with each lipid. The literature describes how the stabilizing mode of each stabilizer also depends on the chemical structure of the lipid; this is confirmed also by the typical case of P407. More analytically, the PPO block of P407 presents a lesser affinity for the phytantriol bilayer, due to unfavorable branching of PPO methyl groups on the hydrocarbon chain of the lipid, resulting in a simple adsorption of P407 to the particle surface when compared to GMO [43,44]. We may assume that the methyl groups of the PLMA block may cause similar effects, resulting in different interactions with each lipid and subsequently different physicochemical behavior. This size difference is also obvious in the 8:1:1 ratio, where P407 is present too. Moreover, the double bond presented in GMO lipid may cause stronger van der Waals interactions with PLMA and PPO block, in contrast to the saturated PHYT.

In addition, the particle size was found to be dependent on the stabilizer amount. The higher polymer concentration is able to provoke a greater reduction of the interfacial tension between molten lipid and water [45], giving nanoparticles of smaller particle size, as the total stabilizer concentration (PDMAEMA-b-PLMA or PDMAEMA-b-PLMA:P407 mixture) is being increased, except from the PHYT:PDMAEMA-b-PLMA 9:3 system. 

As far as the size distribution is concerned, the GMO systems exhibited medium values of PDI (<0.386) and were proven to be more homogenous compared to the PHYT ones. All systems presented medium to high positive values of ζ-potential (Table 2), because the majority of the amino groups of PDMAEMA block are protonated in the pH values of the dispersions (pH ≤ 6). The GMO ternary system 8:1:1 presented less positive value of ζ-potential. It is possible the longer hydrophilic block P407 can hide some charged amino groups of the shorter PDMAEMA block. Moreover, the acidification used during the preparation of GMO:PDMAEMA-b-PLMA 9:1 and 9:3 systems may explain the higher positive values of ζ-potential compared to the PHYT ones (more amino groups being charged).

#### 3.1.2. The Effect of pH on the Physicochemical Behavior of the Nanosystems

**Figure 2:**
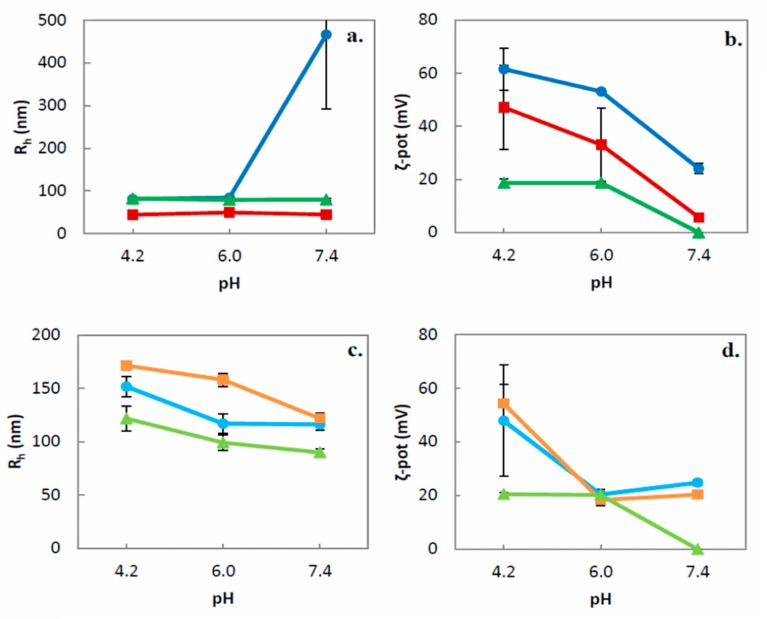
Hydrodynamic radius (*R_h_*, nm) of **a.** GMO and **c.** PHYT nanosystems, as well as ζ-potential (mV) of **b.** GMO and **d.** PHYT nanosystems depending on the pH of the dilution medium (●: Lipid:PDMAEMA-b-PLMA 9:1, ■: Lipid:PDMAEMA-b-PLMA 9:3, ▲: Lipid:PDMAEMA-b-PLMA:P407 8:1:1).

While the PDMAEMA-b-PLMA possesses pH-responsive properties, the pH alterations of the environment are expected to influence the physicochemical behavior of the prepared nanosystems (Figure 2). The physicochemical behaviour of the nanosystems was monitored at three different pH values. Apart from HPLC grade water (pH = 6.0), which is the containing solvent of all the dispersions, PBS buffer with pH = 7.4 was used to verify whether the nanosystems would be stable during their potential administration in physiological blood conditions. Citrate buffer with pH = 4.2 was used to investigate the response of the nanosystems to acidic pH values. For example, endosome lumens exhibit pH values of 4.5–5.5 [9], so the response of the nanoparticle to such acidic conditions would be useful towards endosomal escape and targeted drug release [9,24].

Starting from the GMO prepared nanosystems (Figure 2a), the 9:1 and 9:3 systems presented a small decrease of their radius (up to 10 nm) and their PDI (SI Figure S1a) upon decrease of the environmental pH from 6.0 to 4.2, in contrast to the ternary system GMO:PDMAEMA-b-PLMA:P407, where the acid environment led to slight size increase, as well as PDI increase. This difference may be attributed to the different pH used during their preparation, while 9:1 and 9:3 systems are already acidic dispersions. When the pH increased to 7.4, the GMO:PDMAEMA-b-PLMA 9:1 presented phase separation and formation of visible aggregates, as it is reflected by the acute increase of its size and PDI values, in contrast to the 9:3 and 8:1:1 systems. The partial deprotonation of the PDMAEMA block at neutral pH probably results in a hydrophilic-to-hydrophobic transition, eventually inducing particle aggregation of system 9:1. After all, during its preparation, the 9:1 required the highest acidification in order to be stabilized, being more prone to phase separation in pH = 7.4, compared to the rest of the systems. On the other hand, all PHYT systems presented a pH-dependant increase of their size and PDI upon the pH decrease (Figure 2c, SI Figure S1b) because the extended PDMAEMA protonation upon pH decrease causes swelling effects and internal charge repulsions between neighboring protonated amino groups. When the pH < pKa, the DMAEMA moieties are fully protonated, leading to osmotic swelling and extension of the corona chains [21,46]. The exceptions are GMO:PDMAEMA-b-PLMA 9:1 and 9:3 that were already prepared in even lower acidic conditions, namely, pH = 3 and pH = 5, respectively. 

Concerning the ζ-potential measurements, we observed some great differences among the different pH values for all systems (Figure 2b,d). For the 9:1 and 9:3 systems, we observed high positive values of ζ-potential in pH = 4.2 (till +62 mV) that decreased at pH = 6.0 and pH = 7.4. In particular, the 9:1 and 9:3 systems presented more acute transitions between pH = 4.2 and pH = 6.0 than the 8:1:1 system. The pH-dependant transition of the charge is attributed to the PDMAEMA block. The PDMAEMA block, being a weak polybase, behaves as a weak cationic polyelectrolyte under pH changes. PDMAEMA’s tertiary amine groups are fully protonated at acidic pH and partially protonated at neutral pH, yielding to the observed ζ-potential shifts [21,46]. The 8:1:1 system, probably due to the presence also of the non-ionized PEO block, exhibited lower positive values at pH 4.2 and 6.0 (+20 mV) that were close to zero at pH = 7.4. This observation indicates that the charge of the systems is pH-responsive, probably due to it containing PDMAEMA-b-PLMA. 

The pH-responsive charge may be proved to be very useful in the future, upon the intracellular drug delivery of the nanosystems. The high positive surface charge values at acidic environment may induce internalization (cellular uptake) via the negatively charged membranes of pathological tissues with lower values of environmental pH. Moreover, the high positive values presenting the nanosystems are ideal for complexation with nucleic acids towards the creation of gene delivery systems, due to the great proton buffering capacity of PDMAEMA. In addition, the pH-dependent shift in the protonation state of the nanoparticle that was observed can facilitate endosomal escape. In detail, when the pH of the endosome is lowered from 7.4 to about 5.0, the influx of H^+^ and Cl^−^ ions into the endosome is amplified by the absorption of protons by the polyamine. This phenomenon, also known as proton sponge effect, increases the osmotic pressure, leading to disruption of the endosomal membrane and eventually release of the nanoparticle to cytosol [9,24,47].

#### 3.1.3. Stability Assessment of the Prepared Nanosystems over Time

**Figure 3:**
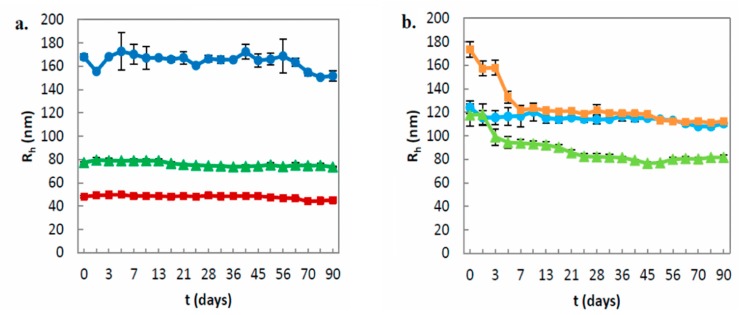
Stability assessment of the size (*R_h_*, nm) of **a.** GMO nanosystems and **b.** PHYT nanosystems over time (●: Lipid:PDMAEMA-b-PLMA 9:1, ■: Lipid:PDMAEMA-b-PLMA 9:3, ▲: Lipid:PDMAEMA-b-PLMA:P407 8:1:1).

Colloidal stability over time was also investigated by measuring the size and size distribution of the nanosystems for a period of 90 days. All GMO dispersions were proved to be stable over time, while there was no significant difference in *R_h_* and PDI values for at least 90 days (Figure 3a, SI Figure S2a). Contrariwise, PHYT systems presented some permanent microscale aggregates, which were stuck around the inner walls of the vials and were not redispersed by hand shaking (Table 1). At the day of aggregate appearance, there was a decrease in *R_h_* and PDI values, which were subsequently stabilized, except for the PHΥT:PDMAEMA-b-PLMA:P407 8:1:1 system, whose characteristics constantly decreased over time (Figure 3b, SI Figure S2b). We may assume that the larger particles of the PHΥT:PDMAEMA-b-PLMA 9:1 and 9:3 systems were aggregated and adhered on the vial, while the smaller ones remained dispersed and stabilized over time. In contrast, the PHΥT:PDMAEMA-b-PLMA:P407 8:1:1 system was proved to be completely instable over time. We should mention that all the presented results of the PHYT systems were collected before the appearance of aggregates.

The colloidal stability of the particles (except for PHΥT:PDMAEMA-b-PLMA:P407 8:1:1) is attributed to the surface adsorption of the polymers. Referring to the P407, its hydrophobic block PPO is solubilized within the non-aqueous domains of the liquid crystals nanosystems and its hydrophilic chains of PEO stretch out the nanoparticle’s surface [4,48]. We suggest that PDMAEMA-b-PLMA, due to its amphiphilic nature, can exhibit stabilizing effects similar to P407. In the pH values of the preparation process (pH ≤ 6.0), the protonated hydrophilic PDMAEMA block can act as a corona, preventing the aggregation of the particles, while the hydrophobic segment PLMA is incorporated into the lipid bilayer. As described in paragraph 3.1.1, we suppose that there are different polymer-lipid interactions, whether the lipid is PHYT or GMO, affecting the ability of the copolymer to stabilize the nanoparticle over time. For example, GMO systems were proven more stable over time than PHYT systems. Apart from their colloidal instability over time, the observed aggregations in PHYT systems also reflect the rapidly changing morphological characteristics. Thus, PHYT systems were not reproduced and not examined by cryo-TEM technique. Taking into account the present physicochemical data as well as their promising pH-responsiveness, different PHYT:PDMAEMA-b-PLMA ratios should be investigated in the future, in order to achieve the stabilization of the PHYT lipid for a longer time. In addition, the combination of PDMAEMA-b-PLMA and P407 successfully stabilized the GMO lipid, but was proven to be unsuccessful in the case of PHYT.

#### 3.1.4. The Effect of the Proteins and the Ionic Strength on the Physicochemical Behavior of the Nanosystems

It is well reported from the previous literature [28,49,50] that the serum proteins may affect intensively the physicochemical behavior of the nanostructures. Indeed, when our dispersions were diluted and incubated with FBS, there were large shifts in their physicochemical characteristics (Table 3). More particularly, the *R_h_* of the PHYT systems was significantly decreased, in contrast to *R_h_* of the GMO systems, which was increased. The PDI of all systems severely increased up to 1.00, reflecting a strict decrease of homogeneity in size. We also observed many peaks at the size distribution graphs, probably due to the formation of supramolecular aggregates of the initial nanostructures with proteins and the other serum components. Concerning the ζ-potential, there was also a decrease of the high positive values to almost neutral or even negative values due to the interaction with the negatively charged proteins.

The neutralization of the charge of all nanosystems reflects complexation phenomena with the serum proteins. However, the different size between GMO and PHYT systems in FBS implies different types of nanosystems–protein interaction. According to the literature, albumin and other serum proteins, like HDL or LDL, can attach onto the cubosomes and disintegrate them, solubilizing the lipids and resulting in small remnant particles with significantly lower size values [51,52]. Our recent study [28] also confirmed the size reduction of liquid crystalline nanoparticles composed of lipid (GMO or PHYT) and P407. More particularly, in the case of PHYT lipid, Azmi et al. [53] have described in detail a decrease of the median particle size of liquid crystalline nanoparticles in the presence of plasma proteins, due to a structural transition of the nanoparticles. Such phenomena may explain the decrease in size of the PHYT systems. The corona effect and steric stabilization of the PDMAEMA-b-PLMA/and P407 was unable to protect the PHYT nanosystems from the albumin present in FBS. Contrariwise, in the case of the GMO systems, the negatively charged serum proteins are electrostatically attracted and complex with the positively charged nanosystems (as seen by the size increase of the nanoparticles and acute decrease of ζ-potential), but without solubilizing them. As a result, the polymeric corona prevented the disintegration of the GMO systems, which may be proved to be an advantage of these systems, regarding their future utilization as therapeutic nanocarriers. As Azmi et al. [54] describe, the absence of plasma-induced degradation of liquid crystalline nanostructures is necessary for their successful pharmaceutical application. 

**Figure 4:**
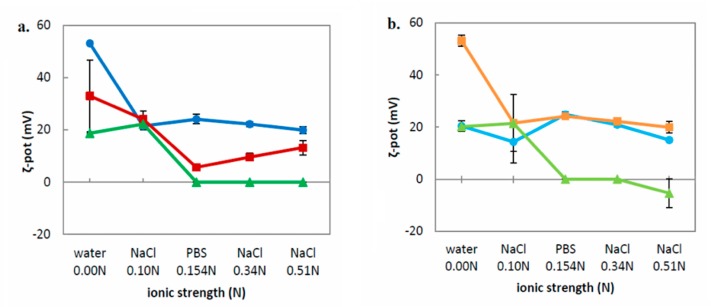
ζ-potential (ζ-pot, mV) of **a.** GMO nanosystems and **b.** PHYT nanosystems diluted in five aqueous media with different ionic strength (●: Lipid:PDMAEMA-b-PLMA 9:1, ■: Lipid:PDMAEMA-b-PLMA 9:3, ▲: Lipid:PDMAEMA-b-PLMA:P407 8:1:1).

After the addition of the NaCl/PBS solution to the nanosystems, they were incubated for 20 min at room temperature before measuring the physicochemical characteristics. Regarding the ζ-potential, the increasing ionic strength resulted in a gradual decrease of the charge. In some nanosystems, we also observed total neutralization of the surface (ζ-potential ca. 0.0 mV) (Figure 4a,b). This phenomenon can be attributed to screening of the positively charged amino groups of the PDMAEMA block by Cl^−^ (NaCl) or phosphate anions (PBS). After 20 min, the increasing ionic strength did not affect the size significantly (SI Figure S3a,c), but it caused some shifts in the PDI values (SI Figure S3b,d). These changes can be attributed to the fact that the increasing ionic strength of the medium may suppress the electric double layer of nanoparticles. The only exception was the PHYT:PDMAEMA-b-PLMA 9:3 nanosystem that presented large shifts of its size and size distribution. However, almost 1 h after adding the NaCl solution to the dispersions, all nanosystems, at ionic strength ≥ 0.34 N presented large aggregates and partial phase separation, unable to be reversed. This phenomenon can be attributed to the screening phenomenon described above, which yielded particle aggregation because the PDMAEMA became more hydrophobic, decreasing the stabilizing efficiency of the hydrophilic corona.

#### 3.1.5. Morphological Characteristics of the Nanosystems as Revealed by SLS/DLS

The *R_g_/R_h_* ratio, calculated by multiangle SLS/DLS experiments, is sensitive to the shape of particles in solution and can be used as a rough estimate of the morphology of the particle. As Burchard [55] described, the *R_g_/R_h_* ratio takes the values of 0.775 for a hard uniform sphere and 1.0 for vesicles with thin walls, while values of 1.3–1.5 indicate a random coil (loose) conformation in the case of macromolecular chains. *R_g_* is a measure of the mass density distribution around the center of mass of the structure and *R_h_* defines the outer hydrodynamic dimensions of the particle. We observed that the majority of the prepared systems presented *R_g_/R_h_* ≤ 0.77 (Table 4), indicating that the nanoassemblies tend to resemble hard uniform spheres. More analytically, as we have recently described [28], the above *R_g_/R_h_* values maybe indicate that the nanosystems possess a confined structure as liquid crystalline particles do, with an almost spherical shape, but not at all vesicle-like morphology, as liposomes do. The only exception was the PHYT:PDMAEMA-b-PLMA:P407 8:1:1 system, which exhibited *R_g_/R_h_* > 1.0. We have to point out that the *R_g_/R_h_* ratio itself cannot fully characterize the morphology. For example, it is reported that the polymeric micelles that can be formed by PDMAEMA-b-PLMA copolymers [21] or other similar block copolymers of PDMAEMA [46] may present *R_g_/R_h_* at ca. 0.775. Thus, we carried out cryo-TEM experiments in order to verify the internal structure, as well as image the particles individually. In addition, when we performed the SLS experiments in acidic environment, we observed some alterations of the *R_g_/R_h_* ratios with no specific trend (Table 4). These alterations may reflect some morphological re-conformation and disturbance of the nanoassemblies, due to a pH-induced transition of the included PDMAEMA chains.

#### 3.1.6. The Effect of Temperature on the Physicochemical and Morphological Behavior of the Nanosystems

**Figure 5:**
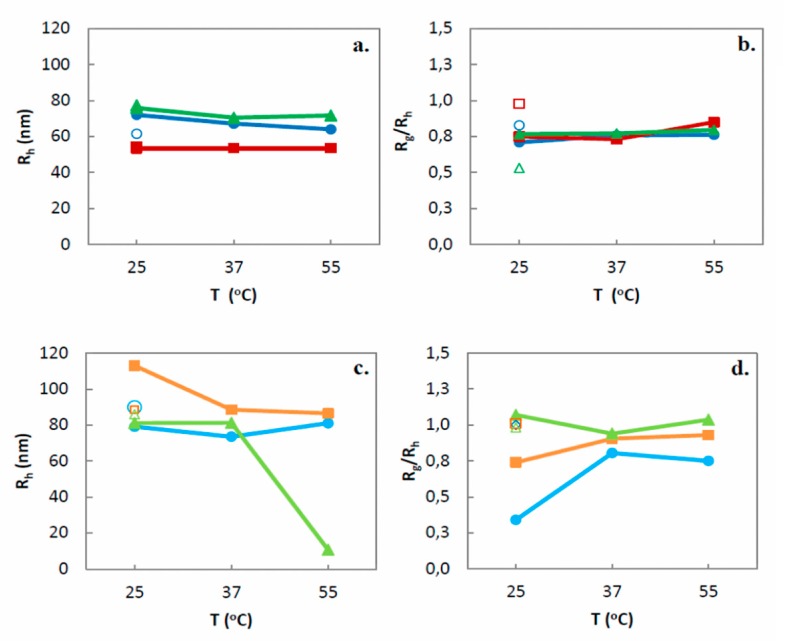
Hydrodynamic radius (*R_h_*, nm) of **a.** GMO and **c.** PHYT nanosystems, as well as *R_g_/R_h_* ratio of **b.** GMO and **d.** PHYT nanosystems depending on the temperature (●: Lipid:PDMAEMA-b-PLMA 9:1, ■: Lipid:PDMAEMA-b-PLMA 9:3, ▲: Lipid:PDMAEMA-b-PLMA:P407 8:1:1).

The temperature dependence of the physicochemical (size and size distribution) and morphological parameters (*R_g_/R_h_* ratio) of the prepared nanosystems was investigated at 25 °C, 37 °C (simulates human body temperature), 55 °C, and 25 °C after a cooling procedure. The empty markers at 25 °C in Figure 5 and SI Figure S4 represent the measured parameters after the cooling procedure.

In particular, GMO systems did not present significant changes of *R_h_* (only GMO:PDMAEMA-b-PLMA 9:1 presented a small irreversible decrease up to 20 nm) (Figure 5a) and PDI values (SI Figure S4a). However, after 37 °C we observed a significant decrease of the scattering intensity, which remained constant during the cooling procedure. While scattering intensity is proportionally related to the mass of the nanoassemblies, we may assume an irreversible decrease of the mass upon temperature increase. The *R_g_/R_h_* ratios of the three GMO systems slightly increased up to 0.85 during heating procedure and were further differentiated during the cooling procedure, reflecting the irreversible temperature transitions of their morphology (Figure 5b). 

PHYT systems presented more obvious alterations upon temperature increase (Figure 5c, SI Figure S4b). PHYT:PDMAEMA-b-PLMA 9:1 presented fluctuations of size and PDI values. The size of the PHYT:PDMAEMA-b-PLMA 9:3 decreased irreversibly at ≥ 37 °C, while its size distribution presented fluctuations. A significant (almost 70 nm), but reversible size decrease, as well as a PDI increase, were performed by the mixed PHYT:PDMAEMA-b-PLMA:P407 8:1:1 system when it was heated above 37 °C. As far as the scattering intensity is concerned, we observed significant lowered irreversible values after heating at 55 °C, for the 9:3 and 8:1:1 systems. The *R_g_/R_h_* ratios of the PHYT:PDMAEMA-b-PLMA 9:1 and 9:3 systems increased up to 1.00 after heating and cooling procedures, reflecting irreversible temperature transitions to a vesicle-like morphology (Figure 5d).

According to the literature, the temperature increase can cause alterations of the hydration degree of the hydrophilic headgroups and the effective volume of the acyl chains of monoglycerides, leading to structural transitions of the liquid crystalline domains [48,56,57]. For example, as described by de Campo et al. [48], monolinolein (MLO)-based liquid crystalline nanoparticles, upon heating, perform reversible structural transformations, attributed to the fact that the nanoparticles expel water upon heating (deswelling/shrinkage) and take up water again upon cooling (swelling) in a reversible way, termed as “breathing mode”. Moreover, P407-stabilized PHYT or GMO nanosystems exhibit reversible temperature-induced structural and size changes [28,58]. 

More analytically, the lattice parameter of all GMO mesophases decreases upon temperature increase and that makes GMO perform typical liquid crystal thermal expansivity in the temperature range of 25–55 °C [56,59], reflected also by a reversible increase of *R_g_/R_h_* ratio values of GMO:P407 systems up to 0.86 [28]. In the present systems with PDMAEMA-b-PLMA, although we observed an *R_g_/R_h_* ratio up to 0.85, indicating structural changes of GMO, the increase of *R_g_/R_h_* ratio was irreversible. Moreover, the heating of the PHYT:P407 systems from 25 °C to above 50 °C results in the reversible transformation of cubic phase to inverse micellar solution [60], associated with a reversible increase of *R_g_/R_h_* ratio values >1.0 [28]. The PHYT systems containing the PDMAEMA-b-PLMA exhibited an increase of *R_g_/R_h_* ratio up to 1.00, probably indicating the aforementioned phase transition, but, similar to the GMO systems, the observed alteration of *R_g_/R_h_* ratio was also irreversible.

We should mention that, opposite to the P407, the use of the PDMAEMA-b-PLMA copolymer as stabilizer provoked non-reversible physicochemical and morphological alterations during heating/cooling procedures, as illustrated by the empty markers in Figure 5. PDMAEMA is thermoresponsive in water solutions, exhibiting LCST in the range 40–50 °C [61,62]. As Chrysostomou and Pispas [21] describe, PDMAEMA above its LCST (>40 °C) becomes more hydrophobic, due to the destruction of the existing hydrogen bonds between PDMAEMA chains and water, resulting in PDMAEMA block shrinkage. The PDMAEMA-b-PLMA employed in this study has been characterized with partial irreversibility upon temperature increase [21]. We suppose that the above described polymer transition, taking place into the microenvironment of our nanosystems, may be responsible for their temperature-induced alterations, due to polymer-induced re-conformations within the nanoparticle’s internal structure. Moreover, the temperature-induced shrinkage of the polymer is maybe responsible for the size decrease of the whole nanoparticles that we observed, due to overall shrinkage of the PDMAEMA corona. We should note that the properties of the polymer, such as the temperature-responsiveness and its partial irreversibility, have been transmitted to the nanosystem. While PLMA exhibits high deformability, due to its low glass transition temperature (T_g_ ca. −53.8 °C) [21,63] it is impossible to influence the nanosystems at the studied temperatures.

### 3.2. Cryo-Transmission Electron Microscopy (Cryo-TEM) Results

**Figure 6:**
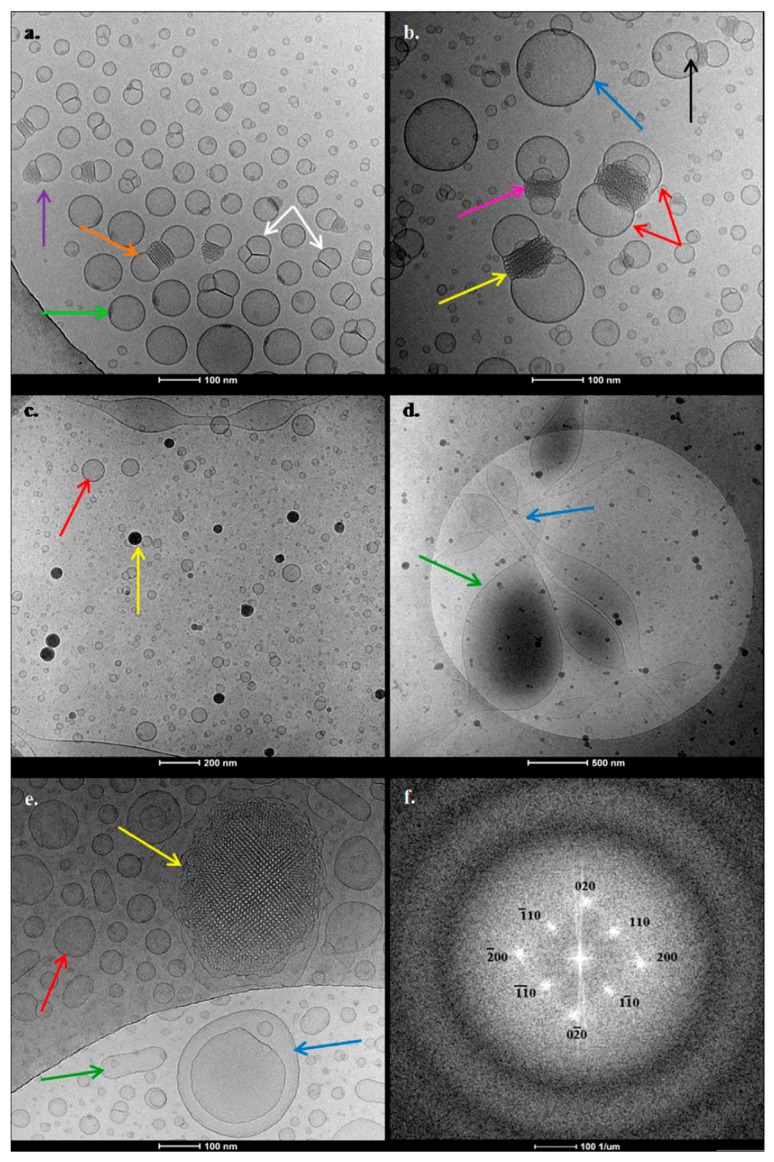
Cryo-TEM images of GMO:PDMAEMA-b-PLMA 9:1 (**a**,**b**), GMO:PDMAEMA-b-PLMA 9:3 (**c**,**d**), GMO:PDMAEMA-b-PLMA:P407 8:1:1 (**e**) and (**f**) FFT pattern from GMO:PDMAEMA-b-PLMA:P407 8:1:1 particle (yellow arrow) likely with the space group *Im3m*.

Cryo-TEM results (Figure 6, Table 5) provided information about the morphology and the internal structure of the GMO nanosystems. The three samples were directly applied in the cryo-TEM instrumentation, exhibiting the pH conditions that are described in Table 1.

Starting from GMO:PDMAEMA-b-PLMA 9:1, two main different categories of objects (Figure 6a,b) were observed. Their characteristics are summarized in Table 5. In particular, there was a large population of vesicles (Figure 6b, blue arrow) exhibiting no internal structure, coexisting with liquid crystalline confined nanoparticles with an ordered structure (Figure 6b, yellow arrow). The most interesting observation of the GMO:PDMAEMA-b-PLMA 9:1 system is the localization of some organized nanoparticles between two vesicles (Figure 6b, red arrow). We should note that this “mixed” structure, as well as another observed mixed structure, consisted of one nanoparticle attached with only one vesicle (Figure 6a, purple arrow); they are both the result of fusion phenomena between ordered nanoparticles and vesicles. The two vesicles are always diametrically attached at each nanoparticle. The two vesicles are either equally sized, providing to the whole structure a “bow-tie”-like shape (Figure 6a, orange arrow), or not equally sized, providing the whole structure with a “lamp”-like shape (Figure 6b, pink arrow). In some of these mixed structures, we can also observe smaller characteristic intersecting lamellas surrounding the central confined nanoparticle (Figure 6b, black arrow). The nanoparticles located in the middle present highly ordered regular inner structures, tending toward a cubosome-like morphology. Moreover, this complex morphology reveals the distribution of the materials. It is possible that the two vesicles contain a greater amount of lipid and lesser amount of polymer, while in the central nanoparticle a larger percentage of the polymer is accumulated, which is able to stabilize the lipids into a more organized structure. 

Apart from nanoparticle-vesicles fusion phenomena, there were also many fusion phenomena between vesicles, yielding triplicate or double multi-vesicular structures (Figure 6a, white arrows). According to the literature, the fusion between unilamellar vesicles is considered to be the precursor of the cubic structure’s creation. The phase transition from lamellar to reverse bicontinuous cubic phase, through intermediate phases, namely stalks and interlamellar attachments (ILAs), has already been stated by Siegel et al. [64]. The fusion between vesicles (lamellar phases) creates interlamellar attachments via stalk intermediates, which gradually evolve to swollen cubic intermediate phase and eventually to the final highly ordered cubic phase [65,66,67]. Thus, the fusion phenomena between vesicles and nanoparticles, as well as among vesicles that are observed in Figure 6a,b may represent different stages upon the dynamic formation of cubic ordered structures. Another slight detail that we pointed out is a kind of spot, existing on the surface of some vesicles (Figure 6a, green arrow). Maybe this spot is the starting point of the fusion phenomena with other vesicles or particles and the evolution from lamellar to organized cubic phases.

Referring to the GMO:PDMAEMA-b-PLMA 9:3 nanosystem, we can observe completely different phenomena. First and foremost, there is an absence of ordered nanoparticles. There are two different size populations of symmetric vesicles (Figure 6c, red arrow), as described in Table 5. Moreover, we also observed fusiform and bulgy vesicles (Figure 6d, green arrow), coexisting with strand objects (Figure 6d, blue arrow). Last but not least, there were many black objects with strong contrast (Figure 6c, yellow arrow), possessing sizes of about 15–70 nm, but we were not able to verify their inner structure. The black objects resemble to polymeric or even mixed lipid-polymer micelles with spherical morphology. After all, the PDMAEMA-b-PLMA block copolymer is able to self-assemble into micelles in aqueous solutions, which consisted of the hydrophobic PLMA core and the hydrophilic PDMAEMA corona, as reported by Chrysostomou and Pispas [21]. The cryo-TEM results of the GMO:PDMAEMA-b-PLMA 9:3 system can be correlated with its different macroscopic appearance. The less milky, but translucent tint of 9:3 can be attributed to the exclusive presence of the vesicles, because the unilamellar vesicles scatter the visible light resulting in the light-blue opalescence which was observed in the dispersion [42].

We may conclude that the amount of stabilizer plays a key role on the morphology and organization of the mixed systems. According to our results, when the PDMAEMA-b-PLMA percentage increases to 25% w/w (relative to the lipid mass), the ordered structures, which were observed in the 9:1 system, were transformed to vesicular and loose structures. In contrast to GMO-P407-based cubosomes, which can accommodate more than 25 wt.% polymer to lipid [68], GMO:PDMAEMA-b-PLMA-based cubosomes cannot incorporate such large amounts of polymer. We may assume that the higher concentration of PDMAEMA-b-PLMA yields a higher grade of perturbation into lipid membrane and eventually disrupts it to less organized structures. As the literature describes, the increase of the stabilizer amount increases the percentage of vesicular structures [68]. Moreover, the high concentration of the stabilizer may lead to formation of mixed micelles that are smaller than cubosomes, as well as mixed GMO/polymer bilayers, which also sterically stabilize the particles against fusion into the cubic phase [69]. It has also been reported that at high stabilizer concentrations, the lipid can be solubilized into the mixed micelles formed, reducing the percentage of cubic structures [39]. The literature also reinforces our opinion about the presence of micelles (illustrated by black objects) at the GMO:PDMAEMA-b-PLMA 9:3. Micelles from block copolymers exhibit different type of morphology than the empty vesicles of the Figure 6 (red arrows). In other cases of PDMAEMA micelles being illustrated with TEM [21,46], we can observe spherical, dense morphologies and small sizes. According to Chrysostomou and Pispas [21], who studied the morphology of the micelles of the same PDMAEMA-b-PLMA block copolymer (that also used in the present study), they observed spherical dense morphologies of dark colour, illustrating most probably only the dense internal core of PLMA and not the more diffuse corona of PDMAEMA chains. Taking into account the above data, we assume that in contrast to the observed empty vesicles, the observed black objects are micellar mixed structures, composed of a dense internal core of PLMA, where are also solubilised some lipid molecules and surrounded by a diffuse corona of PDMAEMA chains.

Concerning the ternary GMO:PDMAEMA-b-PLMA:P407 8:1:1 system, there were also two main categories of objects. We observe vesicles with no internal structure (Figure 6e, red and green arrows) coexisting with liquid crystalline nanoparticles that exhibited confined, highly ordered, periodical inner structure, resembling cubic phase (Figure 6e, yellow arrow). These organized nanoparticles were also surrounded by a kind of “shell/coating” that consisted of vesicular-like structures. In order to investigate the internal structure of the organized nanoparticles, we obtained fast Fourier transform (FFT) patterns of the TEM images (Figure 6f). The patterns show diffuse peaks of brightness, which proves the ordering of internal structure of the particle indicated in Figure 6e. FFT reflections are likely to correspond to the space group *Im3m* [70,71]. Controlled tilting of samples or cryo transmission electron tomography is required for precise determination. As far as the vesicles are concerned, they presented three different size populations (Table 5), having different shapes. We can see spherical vesicles (Figure 6e, red arrow) accompanied with elongated ones that resemble the shape of bacterial rods (Figure 6e, green arrow). Many fusion phenomena between vesicles are illustrated, as well as nested vesicular structures, where large vesicles accommodate smaller ones (Figure 6e, blue arrow), also known as "pregnant" vesicles [72]. We should note that the presence of P407 in equal ratio to PDMAEMA-b-PLMA provided different conformation at the observed liquid crystalline nanoparticles than the conformation of the respective ones of the 9:1 system. In GMO:PDMAEMA-b-PLMA:P407 8:1:1 system, the nanoparticles were larger than formed by GMO:PDMAEMA-b-PLMA 9:1, exhibiting a larger organized surface, while the vesicles were perimetrically attached, not just in the two edges.

In addition, we should analyze some common points which were presented in all the GMO systems. For example, the vesicular structures, presenting in the all prepared nanosystems may have mixed membranes, composed from both GMO and PDMAEMA-b-PLMA or PDMAEMA-b-PLMA and P407. Taking into account that the PPO block of P407 is capable of inserting within the GMO bilayer [68], we suppose that the PLMA block also perturbs the lipid bilayer. Furthermore, the different sizes of the illustrated structures can be correlated with the observed values of PDI of the three systems (Table 2). Moreover, the preparation method also plays a key role in the vesicle formation. We used the TD method with sonication, applying high shear forces in order to achieve homogenous dispersions. As the previous literature describes, during TD method the liquid crystalline nanoparticles may break down into nonequilibrium vesicular structures, which can subsequently fuse to unstable larger ones. However, the increased temperature during the sonication process is able to transform the vesicles into smaller cubic phase nanoparticles. Thus, the coexistence of the different organized structures is well documented, and it is also considered to provide further stabilization [73] and can explain our results for the 9:1 and 8:1:1 systems. The vesicles that were attached to the organized nanoparticles of 9:1 and 8:1:1 molar ratios are proved to play a critical role upon the stabilization of the cubic phase because they can act as a protecting layer, keeping the cubic nanoparticles dispersed in the aqueous environment [68]. Demurtas et al. [67] also confirm the vesicular caps can prevent the exposure of the hydrophobic parts of the stabilizer or the lipids to the water phase. 

Although the morphological diversity is considered to be a stabilization factor [73], the size distribution as presented in Table 5 should be further improved to one-sized populations with low values of PDI, in order for these nanosystems to be successfully employed as drug delivery nanosystems in the future. We can change the size and size distribution characteristics by modifying the preparation protocol. In our study, where a top down (TD) preparation method along with an intermediate step of thin lipidic film preparation was used, an increase of the input energy can be tried. However, while the energy increase would have the risk of disrupting the observed confined liquid crystalline structure due to an input of excessive energy into the system [44], it should be accurately applied. Thus, the use of another preparation protocol can also be investigated, such as the bottom up method, where liquid precursor mixtures of lipid–ethanol are diluted with aqueous P407 solutions, providing cubosomes with minimal input of energy [39,44,74]. Spicer and Hayden [74], who first introduced this method, described how cubosomes prepared by hydrotrope methods have a more homogenous distribution of the stabilizing polymer (e.g., P407) on their surface during the fragmentation process, when compared to cubosomes prepared by mechanical dispersion, resulting eventually in lower values of PDI. The inclusion of ethanol in the formulation increases the solubility of the lipid and thereby significantly reduces its operative viscosity. As Rizwan et al. [44] described, other hydrotrope solvents apart from ethanol can also be used. Taking into account the already acquired physicochemical and morphological data, additional experiments could be designed, and other preparation protocols could be tried, in order to compare their efficacy, as well as to optimise all the involved parameters, in order to result in the optimal preparation method that is able to provide the desired homogeneity at the presented systems.

### 3.3. X-ray Diffraction (XRD) Results

**Figure 7:**
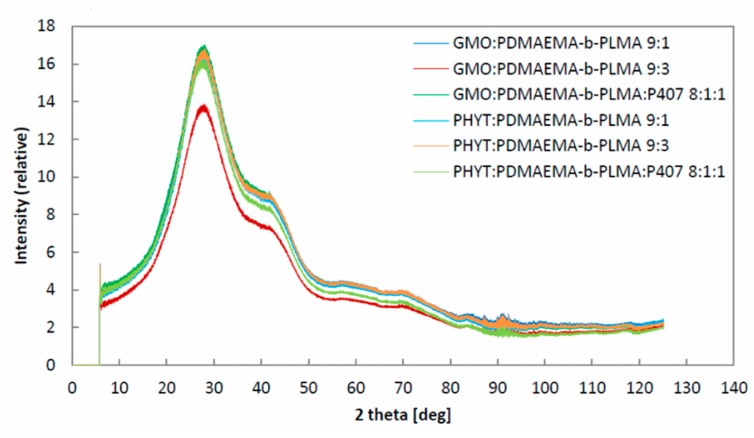
X-ray diffractograms of the prepared nanosystems.

The prepared systems were studied using the same instrumentation as the PHYT/GMO:P407-based nanosystems investigated in our recently published study [28]. All PDMAEMA-b-PLMA-based nanodispersions presented almost the same pattern with the P407-based ones (Figure 7). In particular, there was a strong peak near the latter at the 2D diffraction plots for relative intensity vs. 2θ (Figure 7), implying that PDMAEMA-b-PLMA may behave in a similar way as P407 does. More analytically, in the case of P407-stabilised nanoparticles [28], the observed strong peak can be assigned to a partially ordered structure of P407 in the GMO and PHYT liquid crystalline nanoparticle dispersions. Taking into account that in the present study the use of exactly the same experimental parameters with the previous one resulted in the almost same position and morphology of the peak, we assume that the replacement of the P407 with the PDMAEMA-b-PLMA, as well as the combination of both P407 and PDMAEMA-b-PLMA, did not affect the XRD pattern. It was only the GMO:PDMAEMA-b-PLMA 9:3 system that gave a lower intensity, but in the same pattern. From the XRD results, we assumed that the PDMAEMA-b-PLMA is able to act as a stabilizer, such as P407. However, the different interactions that took place between the different lipids were not revealed by the XRD experiments. Thus, further fluorescence spectroscopy experiments were carried out to highlight the different way of PDMAEMA-b-PLMA interactions between PHYT and GMO nanoparticles, regarding their microenvironment parameters.

### 3.4. Fluorescence Spectroscopy Results

Fluorescence spectroscopy experiments were carried out in order to investigate some critical parameters of the internal nanostructure and microenvironment of the prepared nanosystems. In particular, we measured the micropolarity and the microfluidity of their membranes depending on the medium pH (i.e., 4.2 and 6.0) and the temperature (25 °C and 45 °C). Pyrene was employed as a hydrophobic probe, able to incorporate itself in the hydrophobic domains that were consisted of lipid and PLMA blocks or lipid, PLMA and PPO blocks. This experimental approach can extract some predictive information upon the potential incorporation of low molecular weight hydrophobic drugs.

Fluorescence spectroscopy results from the GMO and PHYT systems are summarized in Table 6 and Table 7, respectively. We observe that the GMO systems presented increased micropolarity (increased values of *I_1_/I_3_*) and almost the same microfluidity (similar values of *I_E_/I_M_*) in comparison to PHYT systems. GMO systems were proved to present more polar bilayers/domains, probably due to the different chemical structures of the lipids (GMO presents a double bond in contrast to the PHYT), that result in different microenvironments, as well as different means of pyrene incorporation within the hydrophobic domains of the nanostructures.

We should note that the different membrane micropolarity verifies the different interactions between PDMAEMA-b-PLMA/PDMAEMA-b-PLMA: P407 mixture with GMO, compared to PHYT lipid. For example, in the case of P407 stabilized cubosomes (classic lipid:P407 formulation), the hydrophobic PPO block exists either at the surface of the cubic phase particles or within the GMO lipid bilayer structure, causing a *Pn3m* (diamond cubic phase) to *Im3m* phase transition (primitive cubic phase), enhancing the water solubilisation capacity of the GMO systems [68,75,76], as well as provoking water swelling into the internal nanostructure [77]. Contrariwise, the PPO exhibits a lower grade of affinity to the PHYT bilayer, due to the unfavorable branching of PPO methyl groups, provoking a simple absorbance on its surface [43,78]. Thus, GMO:P407 bilayers are proved to be more polar that PHYT. We assume that such a different mode of interaction with the lipids may take place in the case of PLMA/PLMA and PPO blocks. The fact that PLMA blocks also have methyl groups, such as the PPO, supports our hypothesis. Moreover, the increased micropolarity of GMO systems may be attributed to the presence of ester groups of the inserted PLMA blocks within the hydrocarbon tail region of the lipid bilayer.

As far as the effect of pH on the microenvironmental parameters is concerned, we observed slight variations up to 0.03 with no specific trend. As PDMAEMA stretches out of the bilayer surface, its pH-induced protonation does affect the inner bilayer. However, there was a small increase (ca. 0.04) of the microfluidity of both groups of systems (with only exception the GMO:PDMAEMA-b-PLMA 9:3 system), at both pH values when the temperature was increased at 45 °C. The temperature-induced PDMAEMA shrinkage and the subsequent contraction of the external polymeric corona may provoke defects at the bilayer, making the membrane more fluid and more permeable to the pyrene. Due to the fact that the PLMA block exhibits high deformability, due to its low glass transition temperature (T_g_ ca. −53.8 °C) [21,63], it is less possible to influence the obtained results. The fluorescence experiments indicate the ability of the nanosystems to accommodate hydrophobic compounds and are useful for the design and development of liquid crystalline nanosystems as future carriers for hydrophobic drugs.

## 4. Conclusions

The stabilization of lipidic liquid crystalline nanosystems by the stimuli-responsive amphiphilic block copolymer PDMAEMA-b-PLMA was investigated in physicochemical and morphological terms. The amount of PDMAEMA-b-PLMA, the presence of the P407 copolymer, and the lipid used (GMO or PHYT) differentiated the resultant nanosystems. More specifically, PDMAEMA-b-PLMA was required to be fully protonated (strong acid dispersion medium) or to be in combination with P407 (ternary system GMO:PDMAEMA-b-PLMA:P407), in order to be able to stabilize GMO lipids. GMO nanosystems were proven to be colloidally stable over time, retaining their physicochemical characteristics. Contrariwise, PHYT systems were less stable over time, revealing different lipid-stabilizer interactions depending on the used lipid. Moreover, the higher amount of PDMAEMA-b-PLMA (i.e., 9:3 ratios) yielded smaller sizes for both the GMO and PHYT systems. All nanosystems exhibited high positive values of ζ-potential, due to charged amino groups of PDMAEMA in their structure. Regarding their pH responsiveness, all nanosystems presented pH-induced alterations of their charge in environments with different pH values. Their positive charge increased in more acidic environment and decreased in neutral pH, although their size and size distribution were not significantly altered. The *R_g_/R_h_* ratio also differentiated in acidic pH, revealing a pH-induced morphological re-conformation. In the presence of proteins, large changes of the physicochemical characteristics of the nanostructures were observed, reflecting complexation and disintegration phenomena depending on the lipid. The increasing ionic strength resulted in gradual neutralization of the nanostructures and subsequent aggregation phenomena. Concerning the temperature effect, we observed some irreversible physicochemical and morphological alterations, probably due to the temperature-induced shrinkage of PDMAEMA. According to Cryo-TEM results, the PDMAEMA-b-PLMA, used in lower amounts (9:1 ratio) or in combination with P407, resulted in the coexistence of different types of objects, including vesicles with no internal structure and confined liquid crystalline nanoparticles with a regular internal structure. The nanoparticles presented vesicle coating of different size and shape. The high amount of PDMAEMA-b-PLMA (9:3 ratio) led to vesicular and loose nanostructures with a low level of organization, indicating that the polymer amount plays a key role in structure organization. Furthermore, fluorescence spectroscopy revealed different values of micropolarity and microfluidity, implying different interaction modes of PDMAEMA-b-PLMA and each lipid. 

In conclusion, the PDMAEMA-b-PLMA at the right concentration, either in combination with P407 or alone, is able to stabilize successfully the GMO liquid crystalline nanoparticles and partially stabilize the PHYT ones. Moreover, the present study proposes the PDMAEMA-b-PLMA as a novel stabilizer for liquid crystalline nanoparticles with advanced stimuli-responsive properties. To the best of our knowledge, this is the first report on liquid crystalline nanoparticles with incorporated polycations of PDMAEMA. On the one hand, the proposed nanosystems retain the advantages of the liquid crystalline internal structure and on the other hand, they exhibit extra pH- and temperature- responsiveness, being ideal for pharmaceutical applications. Their pH-responsive charge conversion can facilitate endosomal escape, uptake by highly negative charged membranes and complexation with nucleic acids for both gene and drug delivery applications. Having completed the aim of this investigation and come to conclusions regarding certain parameters, such as the chosen lipid and the right polymer concentration, our future step is to plan additional experiments, where the best candidates of this class of systems will be studied with other different techniques, so their properties as well as their potential use as drug delivery nanosystems can be further investigated.

## Figures and Tables

**Table 1:** pH value and macroscopical characteristics of the prepared nanoparticle dispersion.

Sample	Weight Ratio	pH of Dispersion	Visual Assessment (t = 0)	Day of Permanent Aggregation Appearance
**GMO:PDMAEMA-b-PLMA**	**9:1**	3.0 *	a	-
**PHYT:PDMAEMA-b-PLMA**	**9:1**	6.0	a	4th day
**GMO:PDMAEMA-b-PLMA**	**9:3**	5.0 *	b	-
**PHYT:PDMAEMA-b-PLMA**	**9:3**	6.0	a	5th day
**GMO:PDMAEMA-b-PLMA:P407**	**8:1:1**	6.0	a	-
**PHYT:PDMAEMA-b-PLMA:P407**	**8:1:1**	6.0	a	2nd day

*: acidification was required in order for homogenous dispersion be achieved, a: homogenous milky opaque dispersion with no lipid aggregates, b: homogenous translucent dispersion with blue opalescence and no lipid aggregates.

**Table 2:** The physicochemical characteristics of the prepared nanosystems in HPLC grade water, on preparation day (t = 0 days).

Sample	Weight Ratio	*R_h_* (nm)	SD _(*Rh*)_	PDI	SD _(PDI)_	ζ-pot (mV)	SD _(ζ-pot)_
**GMO:PDMAEMA-b-PLMA**	**9:1**	84.0	2.5	0.386	0.029	53.1	0.7
**PHYT:PDMAEMA-b-PLMA**	**9:1**	124.1	5.3	0.325	0.026	20.4	1.9
**GMO:PDMAEMA-b-PLMA**	**9:3**	48.2	1.0	0.279	0.003	33.0	13.8
**PHYT:PDMAEMA-b-PLMA**	**9:3**	173.5	6.4	0.523	0.091	18.3	2.1
**GMO:PDMAEMA-b-PLMA:P407**	**8:1:1**	77.3	0.9	0.248	0.019	18.7	0.1
**PHYT:PDMAEMA-b-PLMA:P407**	**8:1:1**	117.8	9.6	0.637	0.033	20.2	0.3

**Table 3:** The physicochemical characteristics of the prepared nanosystems in aqueous and biological medium (fetal bovine serum, FBS).

Sample	Weight Ratio	Medium	R_h_ (nm)	SD _(Rh)_	PDI	SD _(PDI)_	ζ-pot (mV)	SD _(ζ-pot)_
**GMO:PDMAEMA-b-PLMA**	**9:1**	water HPLC grade	84.1	0.2	0.334	0.011	53.1	3.6
		FBS	114.3	32.2	0.930	0.121	−6.1	1.1
**PHYT:PDMAEMA-b-PLMA**	**9:1**	water HPLC grade	120.4	7.6	0.266	0.026	20.4	1.9
		FBS	46.2	3.4	0.867	0.018	2.0	1.0
**GMO:PDMAEMA-b-PLMA**	**9:3**	water HPLC grade	48.2	1.0	0.279	0.003	33.0	0.7
		FBS	129.0	3.7	1.000	0.000	−4.4	2.2
**PHYT:PDMAEMA-b-PLMA**	**9:3**	water HPLC grade	132.9	4.8	0.481	0.043	18.3	2.1
		FBS	71.7	11.3	1.000	0.000	2.1	1.4
**GMO:PDMAEMA-b-PLMA:P407**	**8:1:1**	water HPLC grade	79.0	1.9	0.239	0.003	18.7	0.1
		FBS	103.5	3.4	1.000	0.000	−5.5	0.3
**PHYT:PDMAEMA-b-PLMA:P407**	**8:1:1**	water HPLC grade	99.0	7.0	0.435	0.018	20.2	0.3
		FBS	70.8	3.8	1.000	0.000	0.0	0.0

**Table 4:** The *R_g_/R_h_* ratio of the prepared nanosystems depending on the pH of the dilution environment.

Sample	Weight Ratio	pH	*R_g_*/*R_h_*
**GMO:PDMAEMA-b-PLMA**	**9:1**	6.0	0.71
		4.2	-
**PHYT:PDMAEMA-b-PLMA**	**9:1**	6.0	0.34
		4.2	0.45
**GMO:PDMAEMA-b-PLMA**	**9:3**	6.0	0.75
		4.2	0.77
**PHYT:PDMAEMA-b-PLMA**	**9:3**	6.0	0.74
		4.2	0.63
**GMO:PDMAEMA-b-PLMA:P407**	**8:1:1**	6.0	0.77
		4.2	1.17
**PHYT:PDMAEMA-b-PLMA:P407**	**8:1:1**	6.0	1.07
		4.2	0.83

**Table 5:** Size and morphological characteristics of the existing vesicles of the GMO prepared systems, acquired by Cryo-TEM.

Sample	Size of Vesicles (nm)	Membrane Thickness of Vesicles (nm)
**GMO:PDMAEMA-b-PLMA 9:1**	5–150	3–4
**GMO:PDMAEMA-b-PLMA 9:3**	5–110	3–4
	500–700	4–5
**GMO:PDMAEMA-b-PLMA:P407 8:1:1**	5–100	3–4
	150–400	
	600–1500	

**Table 6:** Fluorescence intensity ratios *I_1_/I_3_* (indicating micropolarity) and *I_E_/I_M_* (indicating microfluidity) for a pyrene probe incorporated into the GMO prepared nanosystems.

Sample	Weight Ratio	pH Medium	T (°C)	*I_1_*/*I_3_*	*I_E_/I_M_*
**GMO:PDMAEMA-b-PLMA**	**9:1**	**6.0**	**25**	1.07	0.08
			**45**	0.99	0.12
		**4.2**	**25**	1.04	0.10
			**45**	1.05	0.12
**GMO:PDMAEMA-b-PLMA**	**9:3**	**6.0**	**25**	1.04	0.05
			**45**	1.07	0.05
		**4.2**	**25**	1.07	0.06
			**45**	1.07	0.06
**GMO:PDMAEMA-b-PLMA:P407**	**8:1:1**	**6.0**	**25**	1.04	0.07
			**45**	1.03	0.12
		**4.2**	**25**	1.06	0.06
			**45**	1.01	0.10

**Table 7:** Fluorescence intensity ratios *I_1_/I_3_* (indicating micropolarity) and *I_E_/I_M_* (indicating microfluidity) for a pyrene probe incorporated in the PHYT prepared nanosystems.

Sample	Weight Ratio	pH Medium	T (°C)	*I_1_*/*I_3_*	*I_E_/I_M_*
**PHYT:PDMAEMA-b-PLMA**	**9:1**	**6.0**	**25**	0.88	0.12
			**45**	0.92	0.15
		**4.2**	**25**	0.87	0.09
			**45**	0.89	0.13
**PHYT:PDMAEMA-b-PLMA**	**9:3**	**6.0**	**25**	0.88	0.07
			**45**	0.93	0.10
		**4.2**	**25**	0.89	0.05
			**45**	0.93	0.08
**PHYT:PDMAEMA-b-PLMA:P407**	**8:1:1**	**6.0**	**25**	0.88	0.07
			**45**	0.92	0.10
		**4.2**	**25**	0.88	0.06
			**45**	0.88	0.09

## Supplementary Materials

The following are available online at https://www.mdpi.com/2073-4360/11/9/1400/s1, **Figure S1:** Polydispersity index (PDI) of **a.** GMO nanosystems and **b.** PHYT nanosystems depending on the pH of the dilution medium (●: Lipid:PDMAEMA-b-PLMA 9:1, ■: Lipid:PDMAEMA-b-PLMA 9:3,▲: Lipid:PDMAEMA-b-PLMA:P407 8:1:1)., **Figure S2:** Stability assessment of Polydispersity index (PDI) of **a.** GMO nanosystems and **b.** PHYT nanosystems over time (●: Lipid:PDMAEMA-b-PLMA 9:1, ■: Lipid:PDMAEMA-b-PLMA 9:3,▲: Lipid:PDMAEMA-b-PLMA:P407 8:1:1)., **Figure S3:** Hydrodynamic radius (*R_h_*, nm) of **a.** GMO and **c.** PHYT nanosystems, as well as Polydispersity index (PDI) of **b.** GMO and **d.** PHYT nanosystems in five aqueous media with different ionic strength (●: Lipid:PDMAEMA-b-PLMA 9:1, ■: Lipid:PDMAEMA-b-PLMA 9:3,▲: Lipid:PDMAEMA-b-PLMA:P407 8:1:1)., **Figure S4:** Polydispersity index (PDI) of **a.** GMO nanosystems and **b.** PHYT nanosystems vs. temperature (●: Lipid:PDMAEMA-b-PLMA 9:1, ■: Lipid:PDMAEMA-b-PLMA 9:3,▲: Lipid:PDMAEMA-b-PLMA:P407 8:1:1)., 2.2.3. Physicochemical and morphological characterization of the prepared liquid crystalline dispersions
