# Supplementary material for: Stimuli-Responsive Lyotropic Liquid Crystalline Nanosystems with Incorporated Poly(2-Dimethylamino Ethyl Methacrylate)-b-Poly(Lauryl Methacrylate) Amphiphilic Block Copolymer
Source: Polymers (Basel). 2019 Aug 26;11(9):1400. doi: 10.3390/polym11091400 (PMC6780812; doi:10.3390/polym11091400)
Supplement: Supplementary file 1 [file polymers-11-01400-s001.pdf]

Supporting Information

Stimuli-responsive lyotropic liquid crystalline nanosystems with incorporated poly(2-dimethylamino ethyl methacrylate)-b-poly(lauryl methacrylate) amphiphilic block copolymer

Maria Chountoulesi [1], Natassa Pippa [1,2], Varvara Chrysostomou [1,2], Stergios Pispas [2,*], Evangelia D. Chrysina [3], Aleksander Forys [4], Lukasz Otulakowski [4], Barbara Trzebicka [4] and Costas Demetzos [1,*]

[1] Section of Pharmaceutical Technology, Department of Pharmacy, School of Health Sciences, National and Kapodistrian University of Athens, Panepistimioupolis Zografou 15771, Athens, Greece; mchountoules@pharm.uoa.gr (M.C.); natpippa@pharm.uoa.gr (N.P.); chrisostomou.varvara@gmail.com (V.C.); demetzos@pharm.uoa.gr (C.D.)

[2] Theoretical and Physical Chemistry Institute, National Hellenic Research Foundation, 48 Vassileos Constantinou Avenue, 11635 Athens, Greece; natpippa@pharm.uoa.gr (N.P.); chrisostomou.varvara@gmail.com (V.C.); pispas@eie.gr (S.P.)

[3] Institute of Biology, Medicinal Chemistry and Biotechnology, National Hellenic Research Foundation, 48 Vassileos Constantinou Avenue, 11635 Athens, Greece; echrysina@eie.gr (E.D.C.)

[4] Centre of Polymer and Carbon Materials, Polish Academy of Sciences, 34 ul. M. Curie-Skłodowskiej, Zabrze, Poland; aforys@cmpw-pan.edu.pl (A.F.); lotulakowski@cmpw-pan.edu.pl (L.O.); btrzebicka@cmpw-pan.edu.pl (B.T.)

* Correspondence: pispas@eie.gr; Tel.: +30-210-7273824 (S.P.); demetzos@pharm.uoa.gr; Tel.: +30-210-7274596 (C.D.)

a.

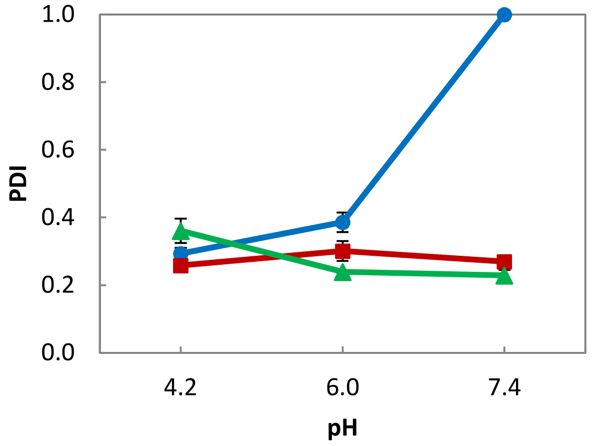

b.

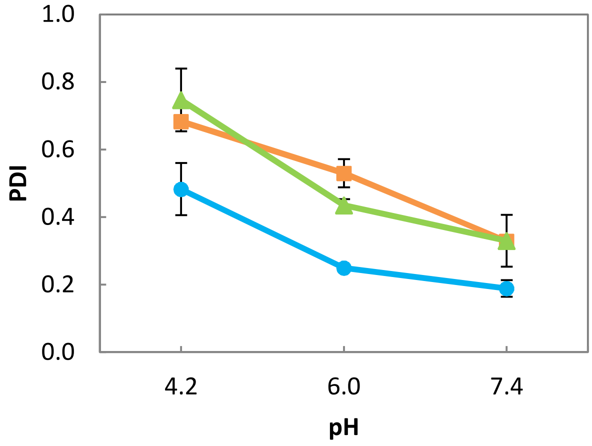

Figure 1. Polydispersity index (PDI) of **a.** GMO nanosystems and **b.** PHYT nanosystems depending on the pH of the dilution medium (●: Lipid:PDMAEMA-b-PLMA 9:1, ■: Lipid:PDMAEMA-b-PLMA 9:3, ▲: Lipid:PDMAEMA-b-PLMA:P407 8:1:1).

a.

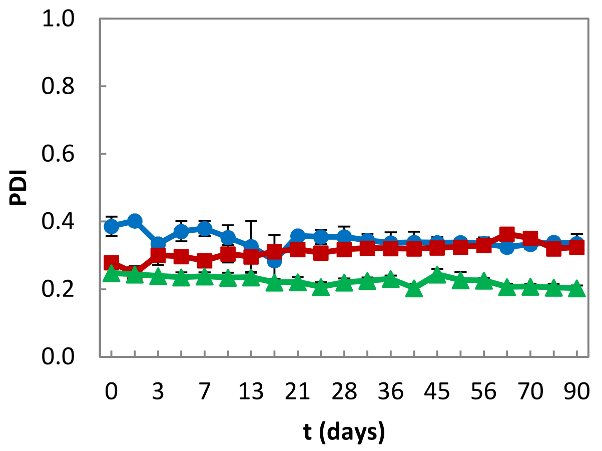

b.

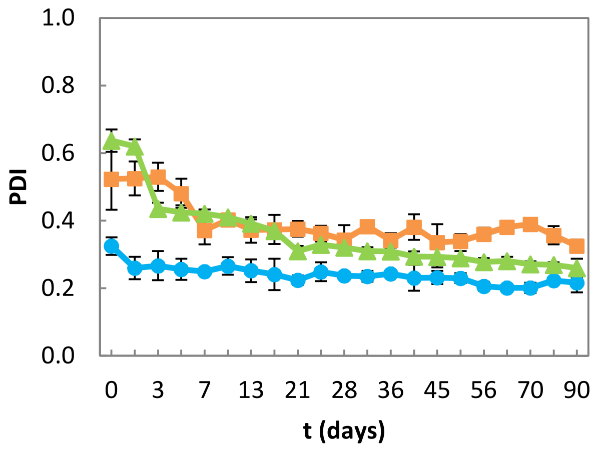

Figure 2. Stability assessment of Polydispersity index (PDI) of **a.** GMO nanosystems and **b.** PHYT nanosystems over time (●: Lipid:PDMAEMA-b-PLMA 9:1, ■: Lipid:PDMAEMA-b-PLMA 9:3,▲: Lipid:PDMAEMA-b-PLMA:P407 8:1:1).

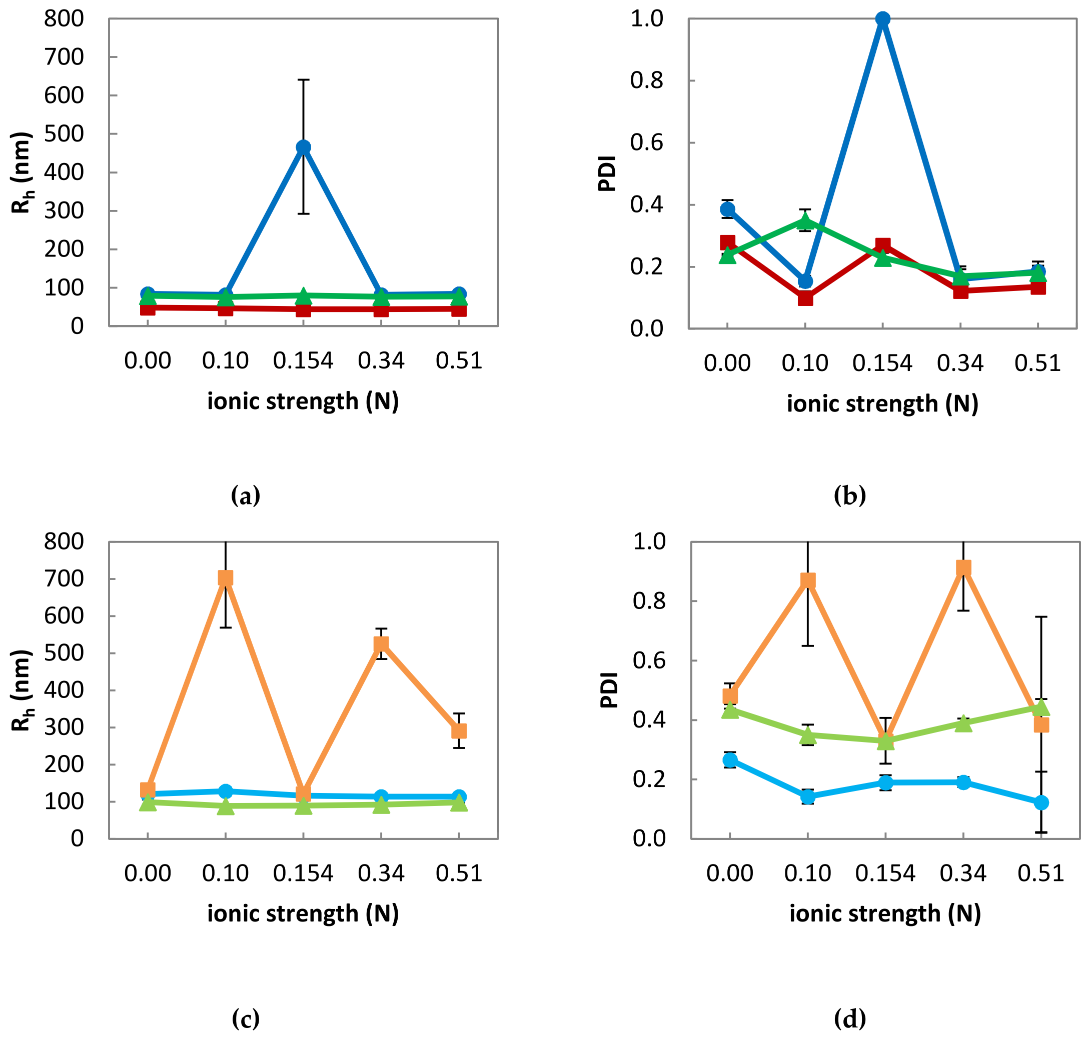

Figure S3: Hydrodynamic radius (R_h, nm) of **a.** GMO and **c.** PHYT nanosystems, as well as Polydispersity index (PDI) of **b.** GMO and **d.** PHYT nanosystems in five aqueous media with different ionic strength (●: Lipid:PDMAEMA-b-PLMA 9:1, ■: Lipid:PDMAEMA-b-PLMA 9:3, ▲: Lipid:PDMAEMA-b-PLMA:P407 8:1:1).

a.

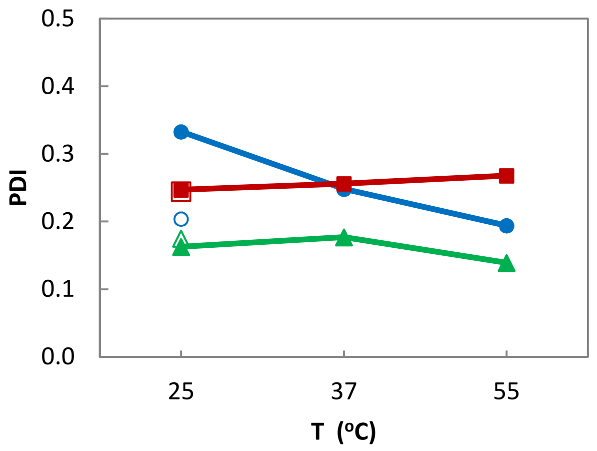

b.

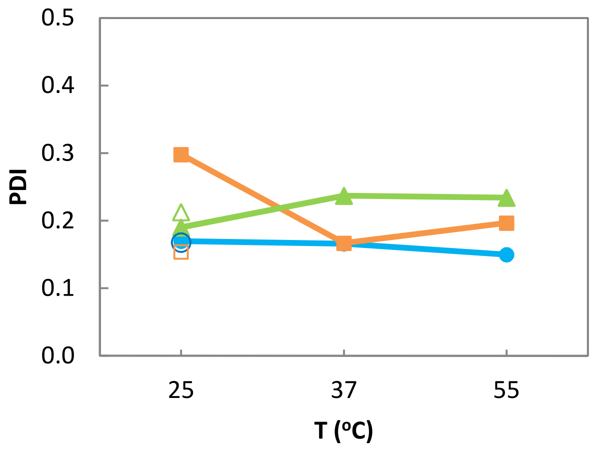

Figure 4. Polydispersity index (PDI) of **a.** GMO nanosystems and **b.** PHYT nanosystems vs. temperature (●: Lipid:PDMAEMA-b-PLMA 9:1, ■: Lipid:PDMAEMA-b-PLMA 9:3,▲: Lipid:PDMAEMA-b-PLMA:P407 8:1:1).

2.2.3. Physicochemical and morphological characterization of the prepared liquid crystalline dispersions

2.2.3.1 Light Scattering Techniques

Dynamic and Static light scattering

The hydrodynamic radius (R_h, nm) and the size polydispersity index (*PDI*) of the prepared nanosystems were measured by dynamic light scattering (DLS). For dynamic and static light scattering measurements, an AVL/CGS-3 Compact Goniometer System (ALV GmbH, Germany) was used, equipped with a cylindrical JDS Uniphase 22 mV He–Ne laser, operating at 632.8 nm,

and an Avalanche photodiode detector. The system was interfaced with an ALV/LSE-5003 electronics unit, for stepper motor drive and limit switch control, and an ALV-5000/ EPP multi-tau digital correlator. Autocorrelation functions were analyzed by the cumulants method and the CONTIN software. Apparent hydrodynamic radii, R_h, at finite concentrations was calculated by the Stokes–Einstein equation:

$$R_h = \frac{k_B T}{6\pi n_0 D} \quad (1)$$

where k_B is the Boltzmann constant, n_0 is the viscosity of water at temperature T, and D is the diffusion coefficient at a fixed concentration. The polydispersity of the particle sizes was given as the μ_2/Γ^2 (PDI) from the cumulants method, where Γ is the average relaxation rate, and μ_2 is its second moment (Chountoulesi et al., 2018; Pippa et al., 2012; Pippa et al., 2013).

The R_h and *PDI* were measured immediately after preparation (t=0 days), as well as for the evaluation of their colloidal stability over time (t=90 days, stored at room temperature). 100μL of aliquots were diluted 30-fold in HPLC-grade water (0.00N and pH=6.0).

The effect of the pH of the medium was investigated by diluting 100μL of aliquots 30-fold in three different pH media, namely HPLC-grade water with pH=6.0, Phosphate Buffered Saline (PBS) with pH=7.4 and Citrate Buffer with pH=4.2. The samples were incubated at room temperature for 20 minutes and the DLS measurements were repeated as described above.

In order to monitor the effect of serum proteins in the physicochemical behavior of the nanosystems, 100μL of aliquots were diluted 30-fold in Fetal Bovine Serum (FBS), incubated at room temperature for 20 minutes and the DLS measurements were repeated as described above (Mohr et al., 2014; Papageorgiou et al., 2018; Chountoulesi et al., 2018).

In order to evaluate the effect of the ionic strength of the medium, 100μL of aliquots were diluted 30-fold in three different ionic strength solutions of NaCl (0.10, 0.34 and 0.51N), as well as in Phosphate Buffered Saline (PBS, 0.154N and pH=7.4, simulating the physiological conditions), incubated at room temperature for 20 minutes and the DLS measurements were repeated as described above (Chountoulesi et al., 2018).

For evaluating the temperature stability of the systems, each measurement was carried out at three different temperature values. The three cell temperatures were 25°C, 37°C and 55°C, using a temperature controlled circulating bath (model 9102 from Polyscience, USA) connected to the measuring vat of the light scattering instrument. Heating and cooling cycles were performed, with equilibration of the systems at intermediate temperatures. An additional measurement was then carried out at 25°C again after the cooling cycle. Equilibration times of 15 min were utilized between measurements at different temperatures.

In order to extract information about the temperature stability and the morphological characteristics of the prepared nanosystems, we carried out simultaneous dynamic and static light scattering measurements in a wide angular range, in order to calculate R_g/R_h ratio. Static Light Scattering experiments at 25°C were also repeated in acidic pH medium (Citrate Buffer with pH=4.2).

Static light scattering was used in order to estimate the radius of gyration, R_g, of the nanosystems via the use of Zimm and Guinier plots. Measurements were made in the angular range of 30° to 150°. Toluene was used as the calibration standard for obtaining absolute values for the scattered intensity. These values were subsequently utilized for the estimation of R_g/R_h ratios, which are indicative of the shape of the nanoassemblies (Chountoulesi et al., 2018; Pippa et al., 2012; Pippa et al., 2013).

Electrophoretic light scattering

The ζ-potential (ζ-pot, mV) of particles was measured immediately after preparation (t=0 days) using Zetasizer 3000HAS, Malvern Instruments, Malvern, UK. 100μL of aliquots were 30-fold diluted in HPLC grade water and ζ-potential was measured at room temperature at 633 nm (Chountoulesi et al., 2018; Pippa et al., 2012; Pippa et al., 2013). Z-potential was also measured in the presence of FBS, PBS, NaCl and Citrate Buffer, as described above. The ζ-potentials were calculated from 7lectrophoretic mobilities (μ_E) using the Henry correction of the Smoluchowski equation:

$$\zeta = \frac{3\mu_E n}{2\varepsilon_0\, \varepsilon_r} \frac{1}{f(\kappa\alpha)} \quad (2)$$

where ε_0 is the permittivity of the vacuum, ε_r is the relative permittivity, α is the particle radius, κ is the Debye length and n is the viscosity of water. The function $f(\kappa\alpha)$ depends on particle shape. While if $\kappa\alpha$>1

$$f(\kappa\alpha) = 1.5 + \frac{9}{2(\kappa\alpha)} + \frac{75}{2(\kappa\alpha)^2} \quad (3)$$

The above function refers to dispersions of this study.

2.2.3.4 Fluorescence spectroscopy

Steady-state fluorescence spectra of the pyrene probe in solutions of the nanostructures were recorded with a double-grating excitation and a single-grating emission spectrofluorometer (Fluorolog-3, model FL3-21, Jobin Yvon-Spex) at two different temperatures 25°C and 45°C. All measurements were performed in two different pH media (HPLC-grade water with pH=6.0 and Citrate Buffer with pH=4.2). The excitation wavelength was λ= 335 nm for pyrene and emission spectra were recorded in the region 355– 630 nm, with an increment of 1 nm, using an integration time of 0.5 s. Slit openings of 2 nm were used for both the excitation and the emitted beams. A stock solution of pyrene 1 mM in acetone was prepared, which was added in the samples in a ratio of 1 μL stock solution per 1 mL of the prepared dispersions. The dispersions containing the probe were left to equilibrate in the dark at room temperature for 24 h before the measurements. The intensity ratio of peak 1 to peak 3, I_1/I_3, serves as a measure of the micropolarity, i.e. a larger I_1/I_3 ratio designates a higher polarity of the medium surrounding the probe. The fluorescence intensities of pyrene excimer I_E and monomer I_M were measured at emission wavelengths 480 and 372 nm respectively, in order to the fluorescence intensity ratio I_E/I_M be estimated. The fluorescence intensity ratio I_E/I_M serves as an index of microfluidity, because the ratio I_E/I_M is proportional to the frequency of collisions of pyrene molecules, namely the ability to form excimers. Larger values of I_E/I_M imply a larger tendency for excimer formation, which can be indicative of a larger microfluidity in the internal domains of the nanosystem (Chountoulesi et al., 2018; Pippa et al., 2012; Pippa et al., 2013).
